# Delayed diagnosis of congenital pulmonary airway malformation in an infant: diagnostic challenges and surgical implications

**DOI:** 10.1093/jscr/rjag924

**Published:** 2026-10-19

**Authors:** Nour Srewell, Youssef Chouman, Yamama Sharief, Mohammed Baraa Meqdad, Mira Hamzeh Rafea, Omar Alsalama, Bushra Alsahan, Mohammd Abdulkader, Sawssan Ali

**Affiliations:** Department of Vascular Surgery, Al-Mouwasat University Hospital, Omar Bin Abdul Aziz Street, Mezzeh, Damascus, Syria; Department of Pediatric Surgery, Children University Hospital, Al-Mouwasat Street, Mezzeh, Damascus, Syria; Department of Pediatric Surgery, Children University Hospital, Al-Mouwasat Street, Mezzeh, Damascus, Syria; Department of Pediatric Surgery, Children University Hospital, Al-Mouwasat Street, Mezzeh, Damascus, Syria; Department of Pediatrics, Children University Hospital, Al-Mouwasat Street, Mezzeh, Damascus, Syria; Department of Pediatric Surgery, Children University Hospital, Al-Mouwasat Street, Mezzeh, Damascus, Syria; Department of Pediatrics, Children University Hospital, Al-Mouwasat Street, Mezzeh, Damascus, Syria; Department of Pediatric Surgery, Children University Hospital, Al-Mouwasat Street, Mezzeh, Damascus, Syria; Department of Pediatrics, Children University Hospital, Al-Mouwasat Street, Mezzeh, Damascus, Syria

**Keywords:** congenital pulmonary airway malformation, pleuropulmonary blastoma, infant, pulmonary cyst, bilobectomy, case report

## Abstract

Although congenital pulmonary airway malformation (CPAM) is usually diagnosed during prenatal screening, delayed presentation may increase diagnostic uncertainty and operative complexity. We report, to our knowledge, the first case of CPAM diagnosed and surgically managed in Syria in a 4-month-old infant presenting with a 1-month history of dyspnea, fever, and poor feeding in the absence of routine prenatal screening. Initial chest radiography, although technically limited, demonstrated a rounded opacity in the right hemithorax, while non-contrast chest computed tomography (CT) revealed multicystic lesions with a hyperattenuating intralesional component, raising suspicion for secondary infection or pleuropulmonary blastoma (PPB). The patient underwent right upper and middle bilobectomy, and histopathological examination confirmed CPAM and excluded PPB. This case highlights the complementary roles of postnatal CT, surgery, and histopathology in confirming CPAM and excluding PPB, particularly when delayed presentation increases operative complexity.

## Introduction

Congenital pulmonary airway malformation (CPAM), formerly known as congenital cystic adenomatoid malformation, is a rare congenital lung malformation resulting from abnormal fetal airway development [1, 2]. Owing to the widespread use of prenatal ultrasonography, most cases are detected before birth, allowing appropriate perinatal counseling and timely postnatal management [1, 3]. Nevertheless, delayed diagnosis may result in recurrent respiratory infections, increased operative complexity, and diagnostic uncertainty, particularly when imaging findings overlap with those of pleuropulmonary blastoma (PPB) [2, 4].

We present a case of CPAM diagnosed and surgically managed in Syria in a 4-month-old infant, highlighting the diagnostic challenges associated with delayed presentation in the absence of routine prenatal screening. This case emphasizes the complementary roles of postnatal computed tomography (CT), surgery, and histopathological examination in confirming the diagnosis and excluding PPB.

## Case report

A 4-month-old male infant was referred to our institution with a 1-month history of progressive dyspnea, fever, and poor feeding. During the preceding weeks, he had been admitted twice to other hospitals because of recurrent respiratory symptoms. At the second admission, he received a blood transfusion for anemia before being referred to the pediatric surgery service with suspected CPAM.

On admission, the patient was hemodynamically stable with a heart rate of 120 beats/min and no signs of respiratory distress or active infection. Laboratory investigations showed no significant inflammatory response (white blood cell count, 9500/μL; C-reactive protein, negative), and hemoglobin was 11.9 g/dL. Initial chest radiography, although technically limited, demonstrated a rounded opacity in the right lung, prompting further evaluation (Fig. 1a).

**Figure 1:**
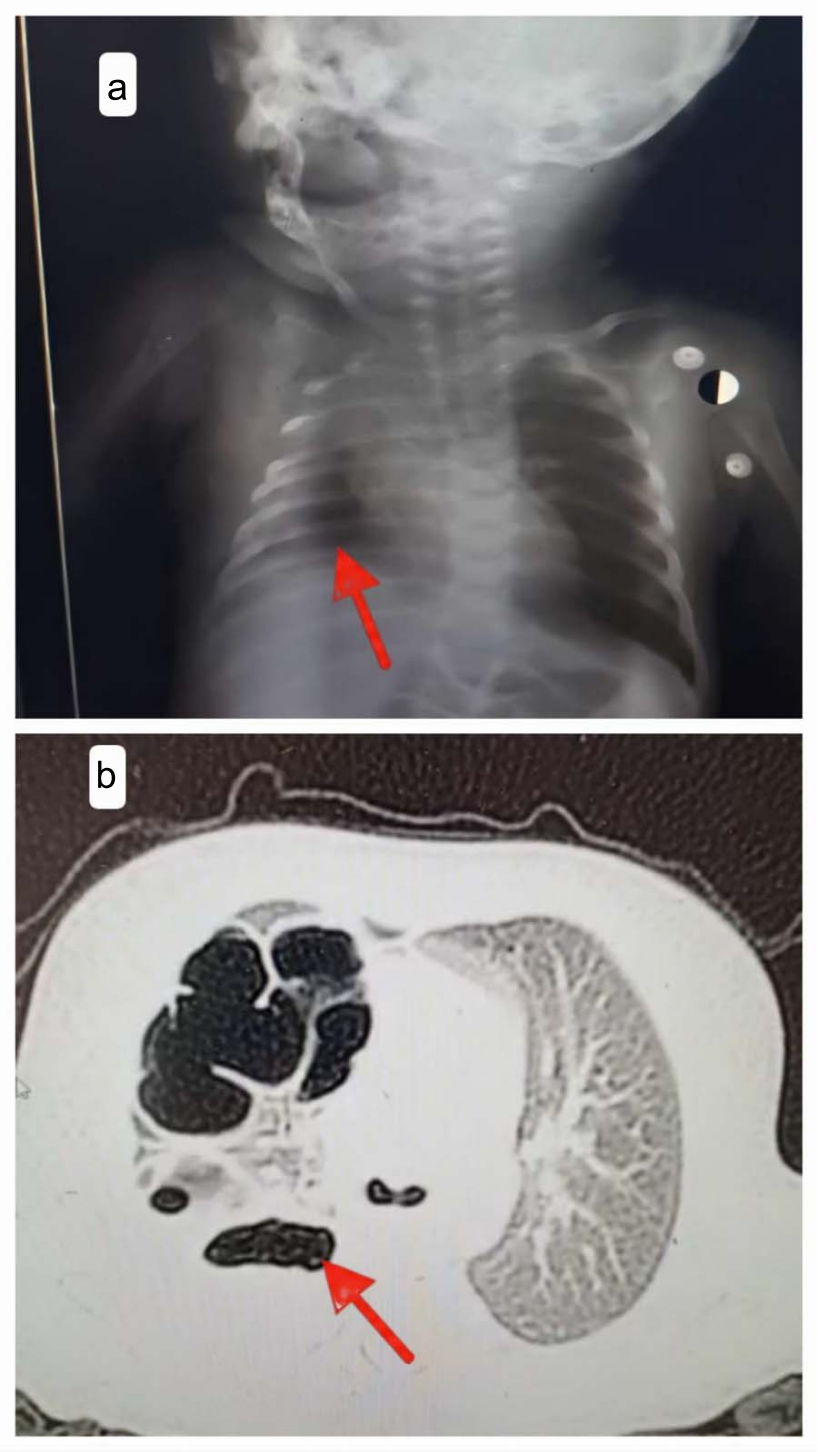
Preoperative imaging findings. (a) Initial anteroposterior chest radiograph demonstrating a rounded opacity in the right lung (arrow). (b) Non-contrast chest CT demonstrating two cystic lesions within the right hemithorax. The inferior lesion contains a dependent hyperattenuating component (arrow), raising concern for superimposed infection and, less likely, pleuropulmonary blastoma.

Non-contrast chest CT demonstrated two cystic lesions within the right hemithorax (Fig. 1b; Supplementary Video 1). The superior lesion arose from the right upper lobe and was completely air-filled, consistent with CPAM. A second adjacent cystic lesion contained a dependent hyperattenuating component, raising concern for superimposed infection and, less likely, PPB arising in type IV CPAM. A small right pleural effusion and residual post-inflammatory scarring were also noted, the left lung and mediastinum were otherwise unremarkable.

Following clinical optimization, the patient underwent right thoracotomy. Intraoperatively, dense pleuropulmonary adhesions and a large cystic lesion originating from the right upper lobe with shared vascular and parenchymal anatomy involving the right middle lobe precluded standard lobectomy. Consequently, a right upper and middle bilobectomy was performed. An iatrogenic air leak from the remaining right lower lobe was repaired primarily, followed by pneumopexy and chest tube placement (Fig. 2).

**Figure 2:**
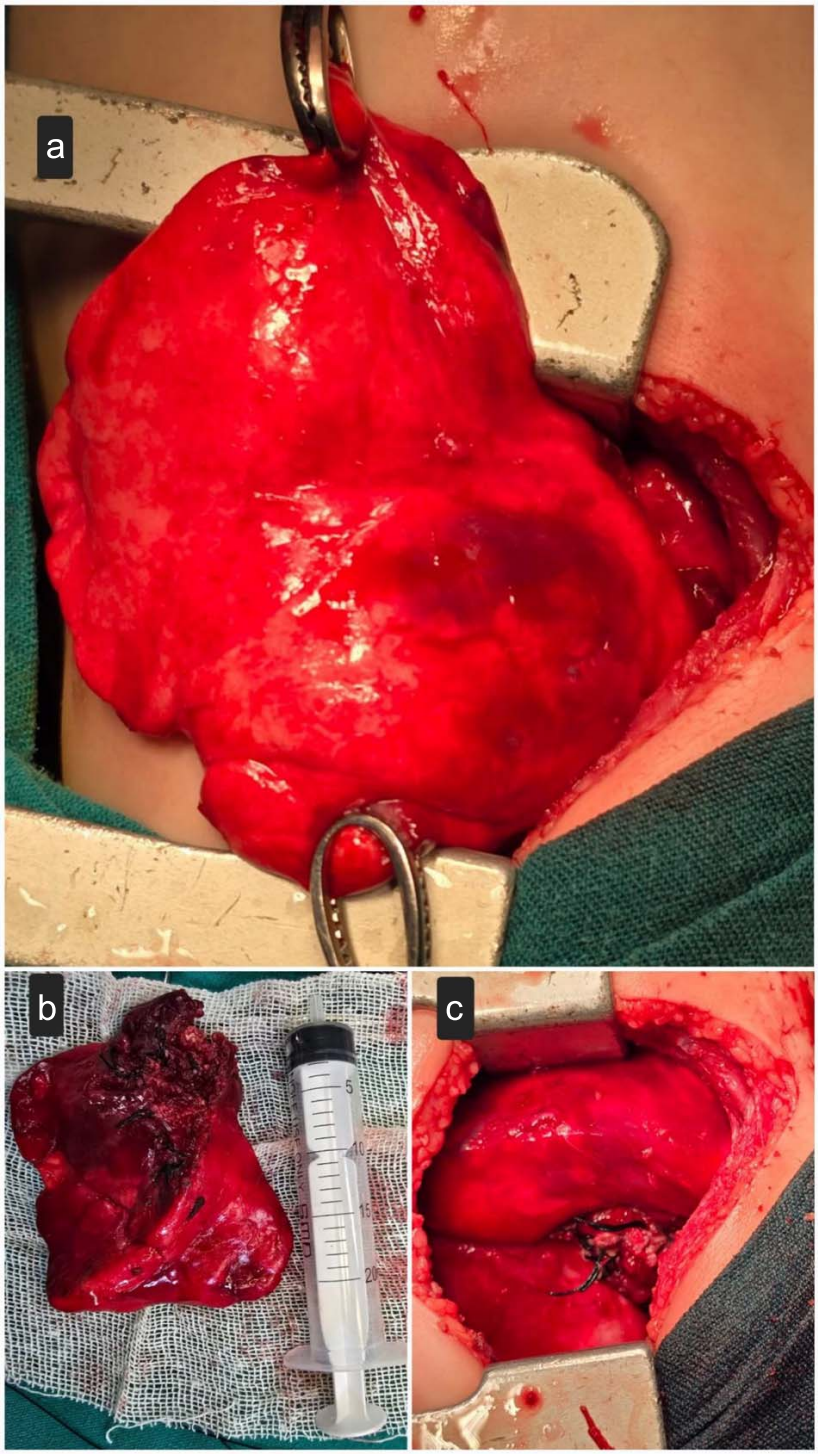
Intraoperative findings. (a) Large cystic lesion involving the right upper and middle lobes. (b) Resected right upper and middle bilobectomy specimen. (c) Remaining right lower lobe following repair of the intraoperative air leak and pneumopexy.

Histopathological examination demonstrated dilated cystic bronchioles with mild emphysematous and chronic inflammatory changes, without evidence of granulomatous disease or malignancy, confirming CPAM and excluding PPB (Fig. 3).

**Figure 3:**
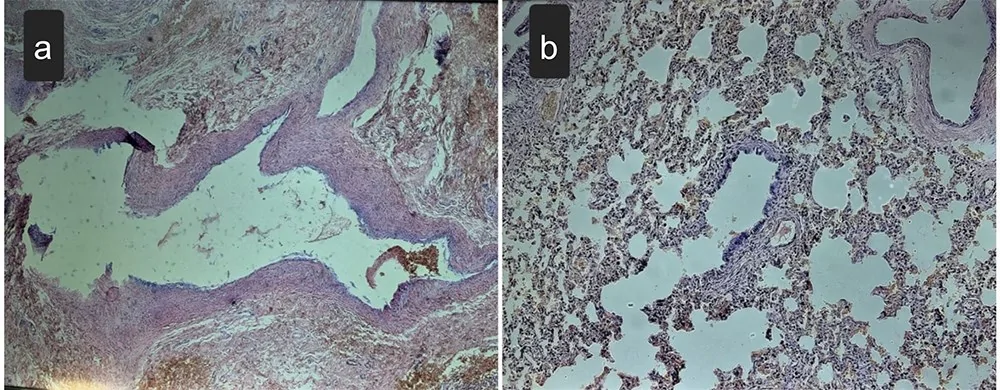
Histopathological examination of the resected lung specimen. (a) Low-power view showing multiple variably sized cystically dilated bronchioles within the lung parenchyma, consistent with congenital pulmonary airway malformation (hematoxylin and eosin stain). (b) Higher-power view demonstrating a cystically dilated bronchiole lined by respiratory epithelium with associated chronic inflammatory infiltrates. No histopathological evidence of malignancy was identified.

The postoperative course was uneventful. The patient required neither mechanical ventilation nor supplemental oxygen after surgery, received postoperative intravenous antibiotics, and remained clinically stable. The chest tube was removed on postoperative day 5, and follow-up chest radiography demonstrated satisfactory expansion of the remaining right lower lobe without postoperative complications (Fig. 4).

**Figure 4:**
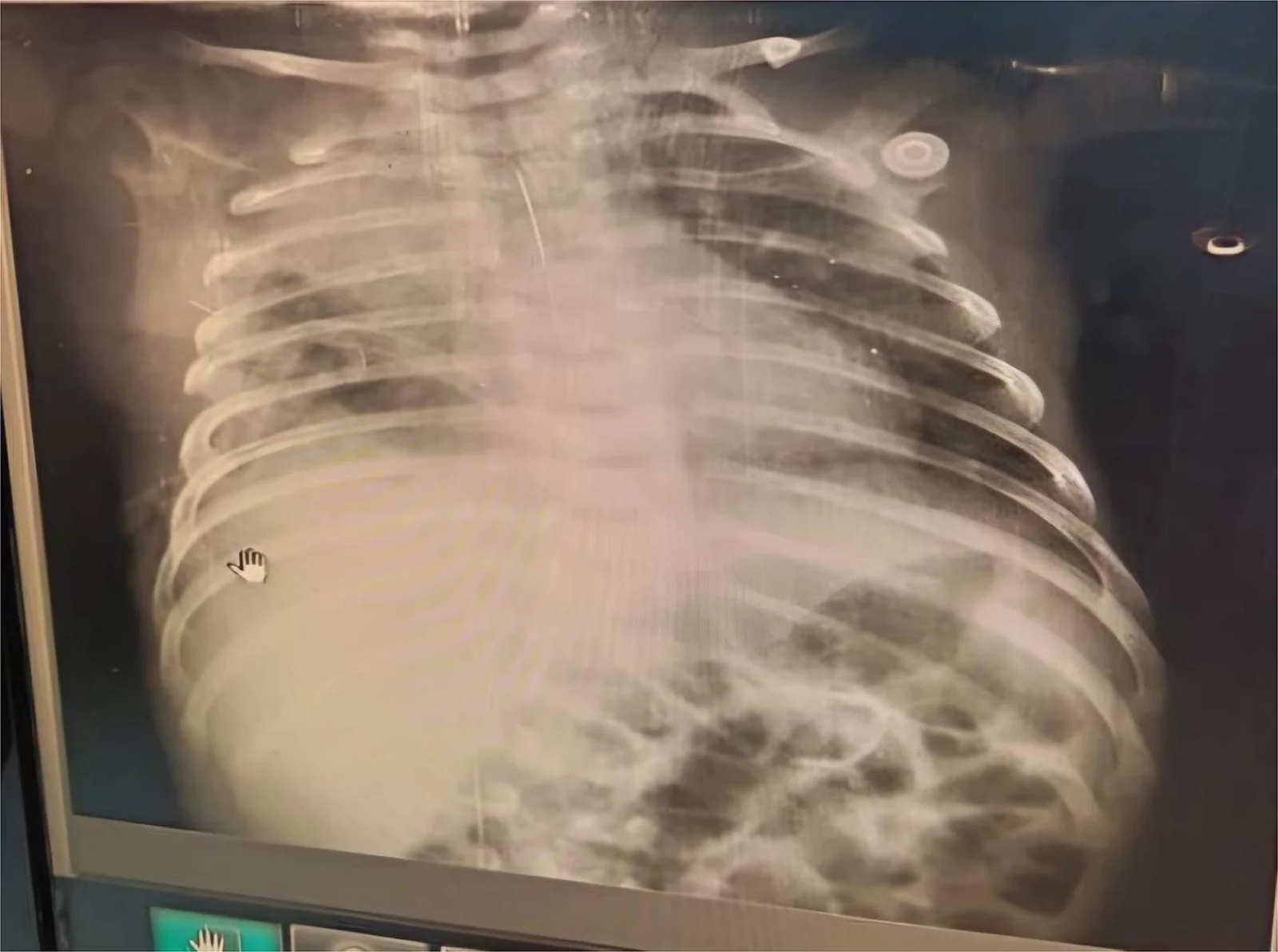
Follow-up chest radiograph obtained on the day of discharge after right upper and middle bilobectomy, demonstrating satisfactory re-expansion of the remaining right lower lobe without radiographic evidence of major postoperative complications.

## Discussion

This case represents, to our knowledge, the first reported case of CPAM diagnosed and surgically managed in Syria and highlights the diagnostic and therapeutic challenges of congenital lung malformations in resource-limited settings. The absence of routine prenatal screening contributed to delayed recognition, allowing a potentially asymptomatic congenital anomaly to progress into recurrent respiratory infections—the most common postnatal presentation of CPAM in infancy—and ultimately a complex surgical condition with challenging radiological findings [1, 2].

CPAM is usually detected prenatally by ultrasonography; however, postnatal CT remains essential for defining lesion extent, identifying complications, and guiding surgical planning. Pederiva *et al.* emphasized the complementary role of prenatal and postnatal imaging in the diagnosis and management of congenital lung malformations [3]. In our patient, CT demonstrated multicystic lesions with a hyperattenuating intralesional component, raising concern for secondary infection or PPB arising in association with type IV CPAM. Differentiating CPAM from PPB remains challenging because of overlapping clinical and radiological features, and Rocha *et al.* highlighted the importance of integrating imaging, histopathology, and genetic evaluation when malignancy cannot be excluded [4].

Distinguishing CPAM from type I PPB may be challenging because of overlapping clinical and radiological features. Although certain prenatal, clinical, and imaging findings may favor one diagnosis over the other, histopathological examination remains the definitive method for establishing the diagnosis and excluding malignancy [5, 6].

The delayed diagnosis contributed to recurrent infections and extensive pleuropulmonary adhesions, necessitating right upper and middle bilobectomy rather than standard lobectomy. Histopathological examination demonstrated cystic bronchiolar dilatation with chronic inflammatory changes and no evidence of malignancy, confirming CPAM and excluding PPB [7]. Early surgical resection during infancy has been associated with favorable outcomes and improved potential for compensatory lung growth [2].

This case emphasizes the complementary roles of CT, surgery, and histopathology in CPAM management and demonstrates how delayed presentation may increase operative complexity while reinforcing the importance of timely diagnosis and intervention.

This case report has been reported in line with the SCARE checklist [8].

## Supplementary Material

VID-20260714-WA0000_1_rjag924
